# Delayed mTOR Inhibition with Low Dose of Everolimus Reduces TGFβ Expression, Attenuates Proteinuria and Renal Damage in the Renal Mass Reduction Model

**DOI:** 10.1371/journal.pone.0032516

**Published:** 2012-03-12

**Authors:** Melania Kurdián, Inmaculada Herrero-Fresneda, Nuria Lloberas, Pepita Gimenez-Bonafe, Virginia Coria, María T. Grande, José Boggia, Leonel Malacrida, Joan Torras, Miguel A. Arévalo, Francisco González-Martínez, José M. López-Novoa, Josep Grinyó, Oscar Noboa

**Affiliations:** 1 Centro de Nefrología, Hospital de Clínicas, Facultad de Medicina, Universidad de la República, Montevideo, Uruguay; 2 Departamento de Fisiopatología, Hospital de Clínicas, Facultad de Medicina, Universidad de la República, Montevideo, Uruguay; 3 Laboratorio de Nefrología Experimental, Departamento de Medicina, Hospital Universitari de Bellvitge, Barcelona, Spain; 4 Departamento de Ciencias Fisiológicas II, Facultad de Medicina, Campus de Bellvitge, Universitat de Barcelona, Spain; 5 Departamento de Anatomía e Histología Humanas, Facultad de Medicina, Universidad de Salamanca, Salamanca, Spain; 6 Departamento de Fisiología y Farmacología, Instituto Reina Sofía de Investigación Nefrológica, Universidad de Salamanca, Salamanca, Spain; Institut National de la Santé et de la Recherche Médicale, France

## Abstract

**Background:**

The immunosuppressive mammalian target of rapamycin (mTOR) inhibitors are widely used in solid organ transplantation, but their effect on kidney disease progression is controversial. mTOR has emerged as one of the main pathways regulating cell growth, proliferation, differentiation, migration, and survival.

The aim of this study was to analyze the effects of delayed inhibition of mTOR pathway with low dose of everolimus on progression of renal disease and TGFβ expression in the 5/6 nephrectomy model in Wistar rats.

**Methods:**

This study evaluated the effects of everolimus (0.3 mg/k/day) introduced 15 days after surgical procedure on renal function, proteinuria, renal histology and mechanisms of fibrosis and proliferation.

**Results:**

Everolimus treated group (EveG) showed significantly less proteinuria and albuminuria, less glomerular and tubulointerstitial damage and fibrosis, fibroblast activation cell proliferation, when compared with control group (CG), even though the EveG remained with high blood pressure. Treatment with everolimus also diminished glomerular hypertrophy.

Everolimus effectively inhibited the increase of mTOR developed in 5/6 nephrectomy animals, without changes in AKT mRNA or protein abundance, but with an increase in the pAKT/AKT ratio. Associated with this inhibition, everolimus blunted the increased expression of TGFβ observed in the remnant kidney model.

**Conclusion:**

Delayed mTOR inhibition with low dose of everolimus significantly prevented progressive renal damage and protected the remnant kidney. mTOR and TGFβ mRNA reduction can partially explain this anti fibrotic effect. mTOR can be a new target to attenuate the progression of chronic kidney disease even in those nephropathies of non-immunologic origin.

## Introduction

In the last few years, mTOR inhibitors such as rapamycin or its derivative everolimus are increasingly used as potent immunosuppressants in renal and cardiac transplant therapy [1].

Chronic allograft nephropathy (CAN) is the main cause of renal allograft loss after one year of transplantation. Despite the impact of modern immunosuppression on reducing acute graft rejection, there has been little impact in long term graft survival [2], [3]. Some investigators propose that mTOR inhibitors can contribute on reducing CAN progression [4].

Although the pathogenesis of chronic damage responsible for CAN is still largely unclear both immune and non-immune mechanisms may participate and they are characterized by an inflammatory response and the subsequent release of profibrotic cytokines and growth factor within the kidney [5]. Chronic interstitial fibrosis, tubular atrophy, vascular occlusive changes and glomerulosclerosis are the common final pathway leading to progressive renal dysfunction and to end stage renal failure [6]. Profibrotic mediators such as TGFβ mainly produced by epithelial cells, may prime their transdifferentiation into fibroblasts and their subsequent activation, directly leading to interstitial fibrosis [7]. TGFβ also stimulates matrix production and reduces its degradation. The severity of tubulointerstitial inflammation and fibrosis have long been considered as crucial determinants in the pathogenesis of renal fibrosis and in long-term prognosis of both human and experimental chronic nephropathies regardless of the initial cause [8], [9].

mTOR is a major downstream component in the phosphoinositide 3-kinase pathway (PI3K), and has emerged as one of the main signalling routes utilized by cells to control their growth, proliferation, differentiation, migration, organization and survival [10].

In addition to lymphocytes, mTOR inhibitors act as anti proliferative for several other cell types such as vascular smooth muscle cells, mesangial, tubular and endothelial cells. Massive urinary protein excretion has been observed in renal transplant recipients with CAN after conversion from calcineurin inhibitors to mTOR inhibitors, especially sirolimus [11]. High range proteinuria has been observed during sirolimus therapy in patients who received sirolimus de novo [12], [13]. Podocyte injury and focal segmental glomerulosclerosis have been related to mTOR inhibition in some patients, but the pathways underlying these lesions remain hypothetic [14], [15].

Controversy exists about the beneficial effects of mTOR inhibition in experimental nephropathies with some reports showing that it may be useful to diminish progression [16], [17] and others reporting increase in proteinuria and aggravation of renal disease [18], [19].

The model of mass reduction with right nephrectomy plus ligation of two branches of the left renal artery (5/6 nephrectomy) has been extensively used to study renal disease progression. Rats with 5/6 nephrectomy develop severe hypertension, proteinuria and progression to end stage renal disease [20]–[22].

The effect of mTOR inhibitors on disease progression in this model also is controversial. Diekmann et al [23] have reported that mTOR inhibitors reduce progression, whereas Vogelbacher et al [19], using the same experimental model, reported that everolimus worsened chronic disease progression.

The aim of this study was to analyze the effects of delayed mTOR inhibition on progression of renal disease in the 5/6 nephrectomy model in Wistar rats, using a low dose of everolimus introduced 2 weeks after nephrectomy and to evaluate its effects on fibrosis mediators as TGFβ.

## Results

### Everolimus treatment decreased proteinuria and albuminuria without changes in blood pressure

Blood pressure, BUN, plasma creatinine, plasma bicarbonate and proteinuria were significantly lower and creatinine clearance was significantly higher in sham group (SG) when compared with control group (CG) and everolimus-treated group (EveG) (table 1). There were no differences in blood pressure, plasma creatinine and creatinine clearance in CG vs EveG. Anyway, EveG showed significant lower proteinuria (142.3±94.8 vs 279.3±125.3 mg/day, p<0.05), protein creatinine ratio (14.45±8.48 vs 28.3±7.47 mg/mg, p<0.05) and urine albumin (6.83±4.6 vs 12.9±4.9 mg/ml, p<0.05) than CG (table 1).

**Table 1:** Weight, blood pressure, renal function, proteinuria and microalbuminuria from animals at week 8 of treatment.

	*SG*	*CG*	*EveG*
	*n = 7*	*n = 11*	*n = 8*
**Weight, g**	360.6±28.4	353.3±48	355.6±26
**Blood Pressure, mmHg**	125 ±15	162±25*	161±25*
**Left Kidney weight, g**	1.4±0.2	2.7±0.8*	2.25±0.36*
**BUN, mg/dl**	21±7	49±40	40±11
**Creatinine_p_, mg/dl**	0.4±0.1	0.78±0.48	0.64±0.13
**Cr Cl, ml/min**	0.52±0.2	0.34±0.11*	0.32±0.12*
**Proteinuria, mg/d**	11.3±13	279.3±125*	142±95*†
**Albuminuria, mg/ml**	wo/d	12.9±4.9	6.83±4.6†
**uPCR, mg/mg**	0.96±1.1	28.3±7.47*	14.45±8.48*†

### Everolimus diminished glomerulosclerosis and tubulointerstitial damage in the remnant kidney

**Figure 1:**
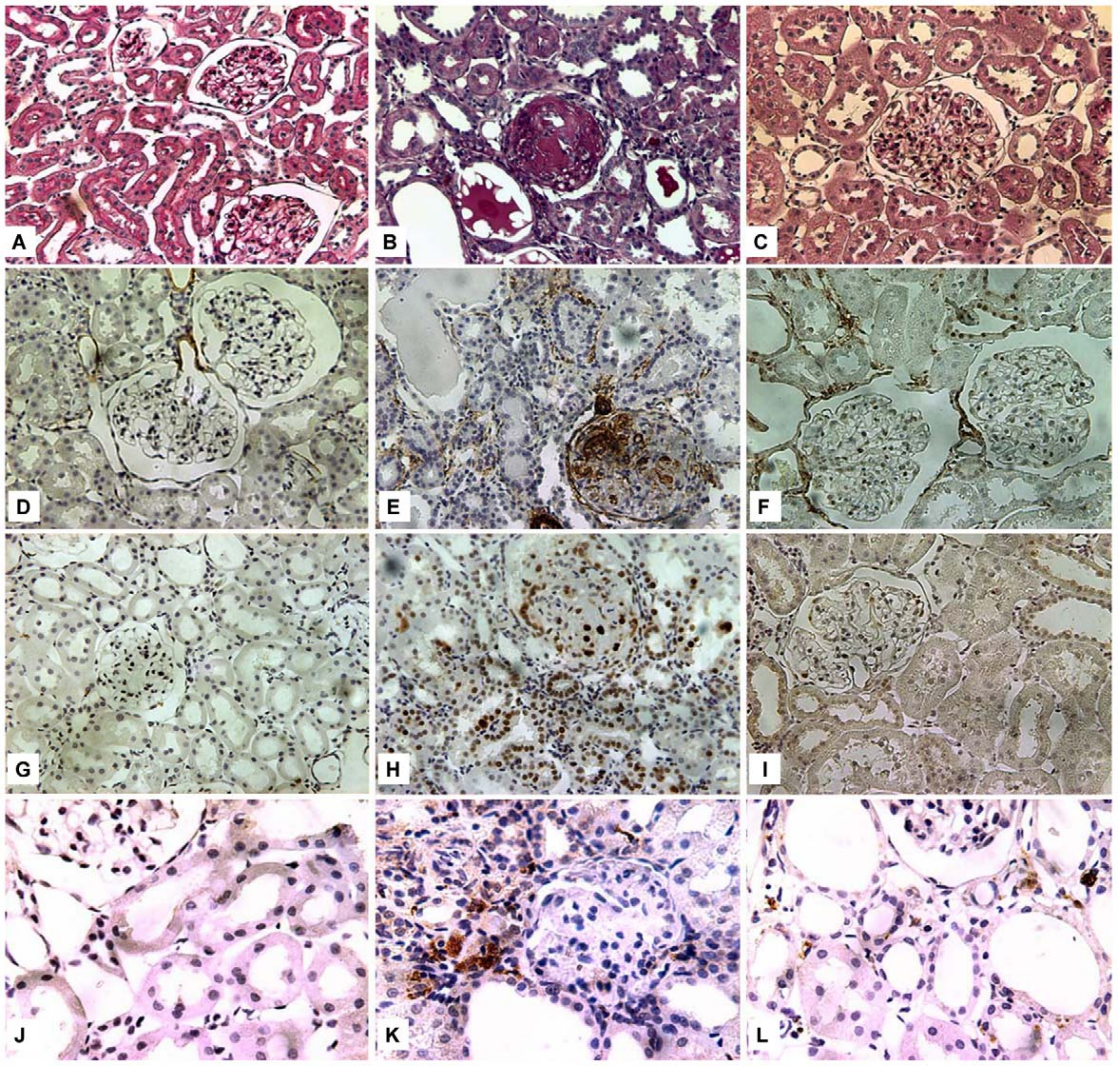
Immunohistochemistry: fibrosis, cell proliferation and inflamation. Representative results from the different groups are shown: Sham Group (A,D,G,J), Control Group (B,E,H,K) and Everolimus Group (C,F,I,L). PAS tinction (A,B,C) (magnification 200×), Immunohistochemistry for α smooth muscle actin (200×) (D,E, F), Glomerular and tubulointerstitial proliferating cell nuclear antigen (PCNA) immunostaining (200×) (G,H,I) and anti CD68 (400×) (J,K,L). Sections were counterstained with eosin.

Histological analysis showed less glomerulosclerosis (GS) in EveG compared with CG when evaluated with PAS stain (0.3±0.43 vs. 1.4±0.73, p<0.05), and with Masson's stain (0.47±0.45 vs. 1.25±0.8, p<0.05). There was also less tubulointerstitial (TI) damage in EveG compared with CG (2.46±1.49 vs. 6.53±1.07, p<0.05) in PAS stain and in Masson's stain (0.1±0.09 vs. 0.24±0.16, p<0.05) (Figure 1 and table 2).

**Table 2:** Histological and immunohistochemistry semiquantitative analysis.

*Stains*		*SG*	*CG*	*EveG*
		*n = 7*	*n = 11*	*n = 8*
**Periodic Acid-Schiff**	G	0.06±0.09	1.40±0.73*	0.30±0.43*†
	TI	0.00±0.00	6.53±1.07*	2.46±1.49*†
**Masson's trichrome**	GS	0.00±0.00	1.25±0.80*	0.47±0.45*†
	TIF	0.00±0.00	0.24±0.16*	0.10±0.09*†
**Sirius Red**	MF	185.6±104.	1153.7±566.2* 0.73*	429.6±283.9*†
	IF	483.5±305.1	2823.3±1424.2 1424.2*	897.4±499.6*†
**α-Actine**	G	0.01±0.02	0.86±0.71*	0.19±0.14*†
	TI	0.00±0.00	2.06±0.55*	1.03±0.52*†
**PCNA**	G	0.05±0.05	6.00±5.37*	2.9±3*†
	TI	0.5±0.5	51.60±29,0*	20±20*†
**CD-68**	I	NA	5.68±3.85	5.05±5.48

Proteinuria is related with the magnitude of tubulointerstitial (TI) damage. When we considered all the animals together, the relationship between proteinuria (mg/day) and TI score, evaluated by Masson's stain, showed a regression coefficient of 0.86, (R2 0.73 p<0.05).

### Everolimus diminished glomerular hypertrophy, mesangial fibrosis and tubulointerstitial fibrosis in the remnant kidney

**Figure 2:**
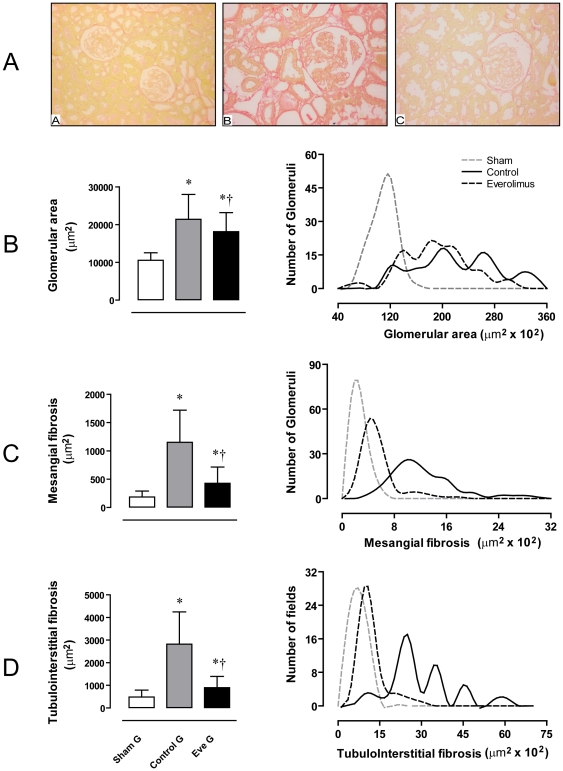
Morphometric analysis of glomerular hypertrophy and kidney fibrosis. Panel A. Representative images from histological sections stained with Sirius red: Sham Group (A), Control Group (B) and Everolimus Group (C) (magnification 200×). Panel B. Glomerular area (media and SD) and frequency distribution diagram of glomerular area. Panel C. Mesangial fibrosis area (mean and SD) and frequency distribution diagram of mesangial fibrosis area in the three groups. Panel D. Tubulointerstitial fibrosis area (mean and SD) and frequency distribution diagram of tubulointerstitial fibrosis area in the three groups. * p<0.05 vs sham group and †p<0.05 vs control group.

Renal fibrosis was also assessed by morphometric analysis of renal tissue stained with Sirius red. Data from morphometric analysis are given as mean ± SD and as frequency distribution, as fibrosis develops in a focal and segmental pattern and its distribution does not show a gaussian pattern. Figure 2A reveals that the CG has a more intense staining with Sirius red than the SG group, being the staining in EveG lower that in CG. Frequency distribution of glomerular area shows that SG have the cross-sectional area of all the glomeruli assessed grouped 10000 µm2, whereas CG and EveG showed a wider distribution, with almost all values over 12000 µm2 (Figure 2b).

The CG showed a higher glomerular area than the SG (21405±6612 vs 10553±1985 µm^2^, p<0.05) whereas treatment with everolimus significantly diminished glomerular hypertrophy (18132±5056 µm^2^) although it remained significantly higher than the SG (p<0.05) (fig. 2b).

Figure 2c reveals that data of glomerular (mesangial) fibrosis in the SG group was grouped around 200 µm2 whereas data of mesangial fibrosis in CG group showed a widespread distribution with higher values, and data from the EveG showed more grouped data around 400 µm^2^. Statistical analysis reveals that the CG showed more mesangial fibrosis than the SG (1153.7±566.2 vs 185.6±104.8 µm^2^ p<0.05). Treatment with everolimus significantly diminished mesangial fibrosis when compared with CG (429.6±283.9 vs 1153.7±566.2. µm^2^, p<0.05).

Figure 2d shows that the changes in tubulo-interstitial fibrosis in the studied groups show changes similar to those observed in glomerular fibrosis. The CG showed significantly more interstitial fibrosis than SG (2823.3±1424.2 vs. 483.5±305.1 µm^2^, p<0.05), and the group treated with everolimus showed lower interstitial fibrosis than CG (897.4±499.6 µm^2^, p<0.05) although it was significantly higher than the SG (p<0.05) (Figure 2).

### Everolimus diminished the expression of α smooth muscle actin and cellular proliferation

We evaluated the effect of everolimus on glomerular expression and TI expression of αsmooth muscle actin. The EveG showed less staining at glomerular level (0.19±0.14 vs 0.86±0.71, p<0.05) and TI level (1.03±0.52 vs 2.06±0.55, p<0.05) when compared with CG (Figure 1 and table 2).

Glomerular and TI PCNA staining was significantly reduced in the EveG compared with CG (2.9±3 vs 6±5.37, p<0.05) and (20±20 vs 51.6±29, p<0.05) respectively (Figure 1).

There were no differences in macrophage infiltration measured by CD68 staining (Figure 1).

### mTOR mRNA increased in the remnant kidney model and it was attenuated with Everolimus treatment

**Figure 3:**
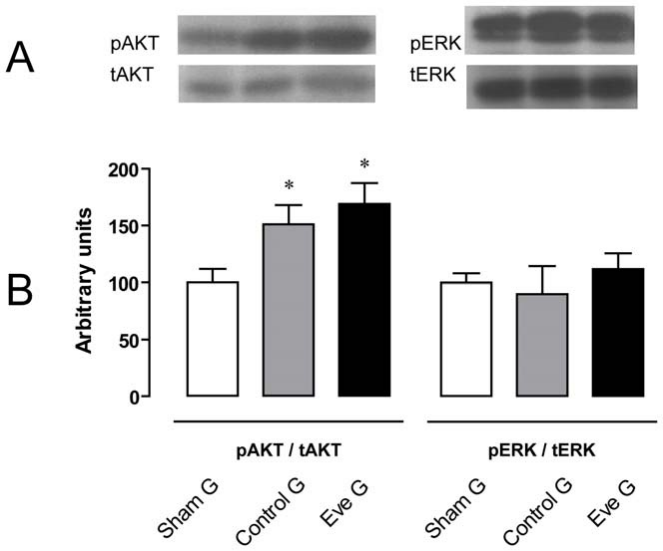
Western blot and protein abundance ratio for pAkt/tAkt and pERK/tERK. A) Inmunoblotting analysis for Total Akt 1–2 and phospho-Akt (Ser473); B) Densitometric analysis of pAkt/total Akt: SG (n = 7), CG (n = 5) and EveG (n = 6); C) Inmunoblotting analysis for ERK 1/2 and p-ERK (E-4), D) Densitometric analysis of pERK/total ERK. SG (n = 7), CG (n = 5) and EveG (n = 6). * p<0.05 vs sham group.

**Figure 4:**
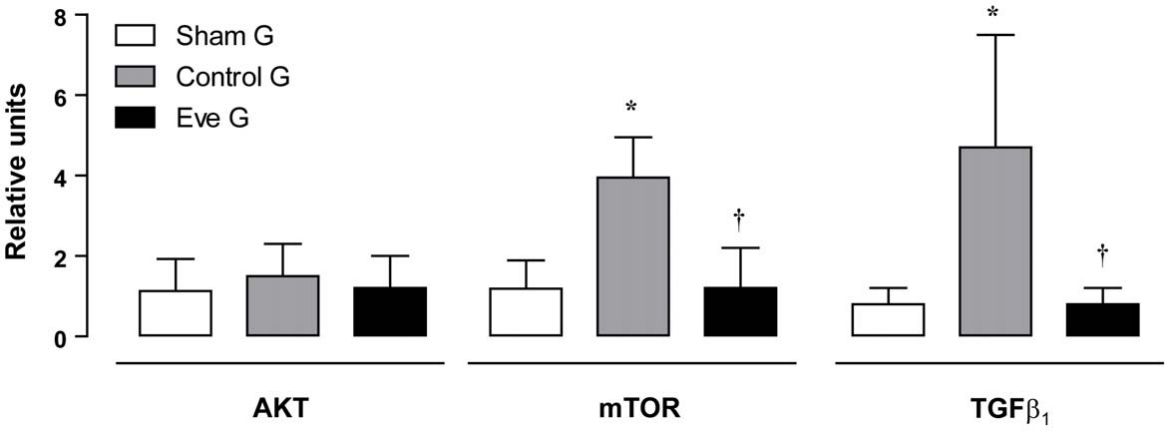
mRNA expression for Akt, mTOR and TGF β. Relative mRNA expression levels of Akt, mTOR and TGF β were assessed by real time quantitative reverse transcriptase-PCR in remnant kidneys of SG (n = 7), CG (n = 5) and EveG (n = 7). *p<0.05 vs sham, **p<0.05 vs CG.

mTOR mRNA is over expressed more than 4-fold in CG when compared with SG (3.95±1 UR vs 1.19±0.7 UR, p<0.05). This increase is blunted in the EveG (1.2±1 UR vs GC, p<0.05) (Figure 3). There were no differences in Akt mRNA among the three groups (SG: 1.13±0.8, CG: 1.5±0.8 and EveG: 1.2±0.3). pAkt/tAkt protein abundance ratio was significantly higher in CG (151.5±17) and EveG (169±18.4) than SG (100±12, p<0.05) but without differences among them (Figure 3 and Figure 4).

### Everolimus blunted the increased expression of TGFβ observed in the remnant kidney model

TGFβ mRNA significantly increased in the CG when compared with SG (4.7±2.8 vs 0.8±0.4 UR, p<0.05). TGFβ in the EveG returned to the SG levels of expression (0.8±0.4 UR, p<0.05 vs GC) (Figure 4).

There were no differences in pERK/ERK ratio among the different groups.

## Discussion

Treatment with everolimus 0.3 mg/kg/day for 8 weeks starting two weeks after 5/6 nephrectomy significantly reduced proteinuria, albuminuria, glomerular and tubulo-interstitial fibrosis, fibroblast proliferation and/or activation, and cellular proliferation.

It also significantly diminished glomerular hypertrophy. Rovira et al showed that sirolimus treatment before 5/6 nephrectomy diminished glomerular hypertrophy but the treatment after nephrectomy was unable to reduce hypertrophy [24]. As far as we know this is the first evidence that everolimus given after 5/6 nephrectomy reduced glomerular hypertrophy, one of the main contributors to glomerulosclerosis in this model [24].

As the fibrosis process develops in a focal and segmental pattern we decided to show the frequency distribution of fibrosis areas in the tissue (panels b, c and d, figure 2). These graphics allows us to visualize the magnitude of fibrosis in the different experimental groups.

There were no differences in renal function between CG and EveG, probably related with the limited follow up time in our model. Other authors using a rat model of polycystic kidney disease were able to show preservation of renal function after prolonged treatment with rapamycin [25].

We found a fourfold increase in mTOR mRNA expression in the remnant kidney model compared with SG. Treatment with everolimus significantly prevented this increase. We can speculate that this inhibition of mTOR mRNA is related with the known transcriptional downregulation effect of mTOR inhibition. In this way mTOR inhibitors prevented the production of new mTOR, diminishing the amount of mTOR available for new mTOR complex formation.

The increase in mTOR mRNA in the remnant kidney model can play a key role in progression of renal disease. mTOR phosphorylation increases mRNA translation via activation of S6 kinase 1 (S6K1) and inhibition of the eukaryotic initiation factor 4E–binding protein1 (4E-BP1), enhancing protein production and cell proliferation [26], [27].

The well known increase in mRNA TGFβexpression observed in the remnant kidney model [28], [29] is completely blunted by everolimus. These results contrast with nephrotoxicity models in normal rats, in which sirolimus increases TGFβ [30]. TGFβ inhibition can partially explain the observed anti-fibrotic effects of everolimus. These results are in accordance with the referred effects of mTOR inhibition in fibroblast activation by TGFβ [27]. Junaid showed, in the 5/6 nephrectomy model, an increase in mRNA TGFβ and protein abundance for TGFβ by elisa, when compared with the sham group. This increase was blocked with losartan [22].

Recent reports indicate that one of the most relevant noncanonical TGFβ¿ pathways is mTORC1 [31], giving more relevance to our findings that mTOR inhibition can affect TGFβ production and signalling.

We found a significant increase in pAkt/tAkt(308) ratio in the CG, consistent with the stimulation off mTOR signaling pathway and also mTORC2 in this model. Recently Sarbassov showed that prolonged exposition to rapamycin is able to inhibit mTORC2 [32]. We were not able to show this inhibition as the treatment with everolimus didn't modified Akt phosphorilation. One alternative explanation to Sarbassov's findings may be that a prolonged exposition to mTOR inhibitors can reduce mTOR availability by means of mTOR mRNA reduction.

On the other hand, Vogelbacher et al [19] using the same experimental model reported that everolimus (2,5 mg/kg/d) introduced 3 days after nephrectomy worsened chronic disease progression. These results were the consequence of a markedly increased fraction of glomeruli with a defective glomerular architecture in the everolimus group. Everolimus apparently inhibited the glomerular repair reaction via proliferative activity inhibition of the glomerular endothelial and mesangial cells and it was associated with reduced glomerular vascular endothelial growth factor mRNA and protein abundance [19]. A recent review [33] highlighted that rapamycin delays recovery and repair of experimental acute kidney Injury [34] and causes and/or exacerbates delay graft function [35].

We used a dose of everolimus more than 8 folds lower than Vogelbacher et al [19]. Dose election was based in preliminary data using different doses (5, 3, 1 and 0.3 mg/kg/day). The animals treated with higher doses showed higher mortality rate and severe adverse effects: diarrhea, weight loss, arrest in wounds healing, and encapsulated abdominal abscess that were not observed with 0.3 mg/kg/day.

We believe that the earlier introduction of high dose everolimus can not only aggravate a pre existent nephropathy but can also produce a new and more aggressive experimental model of nephropathy. Letavernier et al., reported that high sirolimus levels may induce focal and segmental glomerulosclerosis *de novo* [36]. Our results are in accordance with those reported by other groups working with the same model. Diekmann et al reported that animals treated with sirolimus had less glomerulosclerosis, tubulointerstitial damage and attenuated the increased expression of renal vascular endothelial growth factor [23]. Nakagawa et al. described that everolimus administration suppressed smooth muscle α actin, macrophage infiltration and kidney injury molecule-1expression in the proximal tubules [37]. Torras et al. demonstrated a higher glomerular podocin and nephrin expression and amelioration of glomerular ultrastructural damage in the rapamycin group [15].

Beneficial effects of mTOR inhibitors were also reported in anti-thy1 nephritis [17], in experimental membranous nephropathy [38], in adriamycin induced nephropathy [39], in unilateral obstructive uropathy [40] and in the murine model of renal polycystic disease [41]. In diabetes induced by streptozotocin [16] sirolimus treatment produced a reduction of albuminuria and the expression of renal mTOR and TGFβ.

Our findings provide *in vivo* evidence that everolimus significantly prevented progressive renal fibrosis and protected the remnant kidney in an experimental model of reduced renal mass. In order to obtain these beneficial effects everolimus should be administrated after the acute effects of renal ablation and reparation have taken effect and in a lower dose than previously described.

The mTOR and TGFβ inhibition can partially explain the anti fibrotic effects produced by everolimus.

This study supports that mTOR can be a possible new target to attenuate the progression of chronic kidney disease [4].

## Methods

### Model and experimental design

All the animals were handled according to the Principles of the Laboratory Animal Care (National Institutes of Health, 1985). The experimental protocol was approved by the University Animal Experimental Committee and by the local ethic committee. The animals were housed in individual cages in a constant-temperature room with 12:12-h dark-light and free access to water and food with a normal protein diet (24%). Rats were anesthetized with sodium thiopental 35 mg/kg i/p for all surgical procedures.

Male Wistar rats with an initial body weight of 300±30 g were used for this study. Initially, simultaneous systolic blood pressure and pulse in the awake animal were registered by the tail cuff method using CVMS-20 software (World Precision Instruments, Sarasota, FL, USA).

Rats were randomly assigned to 3 groups. Two of these groups underwent 5/6 nephrectomy by selective ligation of renal artery branches followed by contra lateral nephrectomy. SG underwent abdominal incision and manipulation of both kidneys without excision.

Two weeks after nephrectomy the EveG received everolimus 0.3 mg/kg/day or vehicle for CG, administered by daily gavages during 8 weeks. Dose election was based in a preliminary study using different doses (5, 3, 1 and 0.3 mg/kg/day). The animals treated with higher doses showed higher mortality rate and severe adverse effects: diarrhea, weight loss, arrest in wounds healing, and encapsulated abdominal abscess that were not observed with 0.3 mg/kg/day. Meanwhile, recent reports have described the use of a lower dose of everolimus in order to reduce mortality [42].

Everolimus for oral administration was provided by Novartis Pharmaceuticals, Inc.

### Functional and Histological studies

The rats were assigned to 3 groups: SG (n = 7), CG (n = 11) and EveG (n = 8). After 8 weeks of treatment 24 hours urine collection was performed and simultaneous systolic blood pressure and pulse were registered. The remnant left kidney was immediately perfused with phosphate-buffered saline at 4°C and 4% paraformaldehyde, and stored for renal morphology and immunohistochemistry studies.

Serum creatinine, BUN, plasma bicarbonate, urinary protein, albumin, low weight molecular protein excretion and creatinine clearance were determined.

### Sample storage for RNA and protein extraction purposes

We performed the same experimental procedure with three new groups of animals: SG (n = 7), CG (n = 5) and EveG (n = 7) to obtain renal tissue for real time RT PCR and Western blot. At week 8 remnant kidney was perfussed with cold PBS. The kidney was removed and renal cortex excised, snap-frozen in liquid nitrogen and stored at −80°C until processing.

### Analytical methods

Serum and urinary creatinine were measured by the buffered kinetic Jaffe reaction. BUN was determined by spectrophotometric method, plasma bicarbonate with a blood analyzer (ABL 700, Radiometer, Copenhagen, Denmark) and urinary protein excretion was assayed by the turbidimetric method with sulfosalicylic acid. Albuminuria and low weight proteinuria were assessed by High Performance Liquid Cromatography according to Turpeinen [43].

### Quantification of renal histology

Coronal sections of the kidney were immersion-fixed in paraformaldehyde solution and embedded in paraffin. Light microscopy was performed on 4-µm sections of tissue stained with periodic acid-Schiff's (PAS) and Masson trichrome reagent (MT) to assess GS and TI fibrosis. GS was assessed by semi quantitative analysis of 20 consecutive glomeruli (400×) according to El Nahas et al [44]. GS was defined as glomeruli with sclerosis or mesangial expansion and/or focal hyalinosis with tuft adherence. Glomerulosclerosis was graded from 0 to 4 by a semi quantitative score: grade *0 normal, *1 mesangial expansion/sclerosis involving less than 25% of the glomerular tuft, *2 moderate GS (25%–50%), *3 severe GS (50%–75%), and grade *4 diffuse GS (more than 75%).

TI damage was evaluated using a semi quantitative analysis of 20 cortical fields (200×) according to Veniant el al [45]. Lesions were graded from 0 to 4 according to the area with tubulointerstitial changes (tubular atrophy, casts, interstitial inflammation, and fibrosis).

The score index in each rat was expressed as a mean value of all scores obtained.

All the histological analysis were performed by an observer unaware of the treatment received by each group.

### Morphometric studies

Morphometric studies were performed in sections 5 µm thick and Sirius Red stained. In brief, images were captured at 20-fold magnification using a green optical filter (IF 550) and a high-resolution videocamera (SONY CCD-iris) connected to a light microscope (Nikon, Eclipse 50i). The evaluation and image analysis procedures were performed with specific software [46].(Fibrosis HR. Master Diagnostica. Granada. Spain) as previously reported [47], [48]. As Sirius Red stains collagen fibers, the program automatically transforms color images in 256 grey levels images and quantifies the elements of the image, previously isolated from the background. In the case of glomerular images, it is necessary to split the corpuscular area by indicating in the monitor where the glomerulus is located. Then the program automatically discriminates the area of renal corpuscles which is usually surrounded by a pericapsular coat of fibers that are stained by Sirius Red.

A total of 25 glomerular images and 10 interstitial images random fields of renal slides (n = 5 animals per group) were captured and processed. The values obtained for each image were: a) glomerular fibrotic area or percentage of Sirius red-stained area which is contained in Bowman's capsule, b) renal corpuscular area and c) interstitial fibrotic area, or percentage of tubulointerstitial area occupied by Sirius red staining (excluding glomeruli and big arteries). These values, saved in data ASCII files conveniently labeled, can be exported to any statistical analyses program and calculate several parameters that can express the degree of renal fibrosis and glomerular sclerosis in an objective and quantitative way.

### Quantification of immunohistochemistry

Sections of paraformaldehyde fixed kidney tissues were processed by indirect immune detection technique with Biotin streptavidin amplified detection system (BioGenex, San Ramon, CA, USA) using three primary antibodies: (1) proliferating nuclear cell antigen (PCNA) (Dako Glostrup, Denmark NP047) (dilution 1∶200), as a marker of cell proliferation, (2) α smooth muscle actin as a marker of myofibroblast transformation (Dako Glostrup, Denmark NP025) (dilution 1∶100) and (3) CD68 as a marker of macrophages (Serotec, Oxford,UK MCA341R) (dilution 1∶100). Next, samples were incubated with secondary antibody conjugated with peroxidase, EnVision® (DAKO, Glostrup, Denmark) for 30 minutes and stained with DAB (EnVision®, DAKO®, Glostrup, Denmark). Tissues were counter stained with hematoxilin.

Normal rabbit IgG (Santa Cruz Biotechnology, Santa Cruz, CA, USA) was used as a negative control. Mean score per biopsy was calculated as follows: glomerular PCNA and CD68 as the mean number of positive cells in 20 glomeruli (cells/glomerular cross section) (400×), CD68 and PCNA tubular/interstitial score was obtained as the mean number of stained cells in 20 fields (cells/fields) (200×).

For the evaluation of α-smooth muscle actin each glomerulus and tubulointerstitial field was graded semi quantitatively according to the extent of the staining from 0 (absent) to 4 more than 75% in the glomerular tuft or more than 75% of tubulointerstitial field. Mean scores of 20 glomeruli and 20 fields were calculated. Immunohistochemistry images were acquired (SnapCool-Pro, Nikon®, Tokio, Japon) and digital analysis was performed (ImagePro-Plus, Versión 4.05 Media Cybernetics Bethesda, MD, USA).

### Western Blot Analysis of Akt, phospho-Akt, ERK and phospho-ERK

Tissue protein extracts were homogenized in lysis buffer (25 mM HEPES pH7,5, 150 mM NaCl, 1% Igepal CA-630, 10 mM MgCl2, 1 mM EDTA, 10% glycerol, 10 µg/mL aprotinin, 10 µg/mL leupeptin, 100 mM PMSF, 25 mM NaF, 1 mM Na3VO4) and centrifuged at 14,000 g for 30 min. Supernatant was recovered and proteins were quantified. Western blot analysis was performed as previously described [49]. In brief lysates (60 µg/lane) were loaded onto SDS-polyacrylamide gels, and the proteins were transferred to nitrocellulose membranes (Bio-Rad) by electroblotting. Membranes blocked in TTBS (10 mM Tris pH 7.5, 150 mM NaCl, 0.1% Tween 20 plus 2% bovine serum albumin) were incubated overnight at 4°C, as appropriate, with: anti-Akt1/2 (1∶1000, sc-8312, Santa Cruz Biotechnology, Inc. Santa Cruz, Temecula CA, USA), anti-phospho-Akt (Ser 473) (1∶1000, #9271, Cell Signaling Technologies, Beverly, MA), anti-ERK1 (1∶2000, sc-94, Santa Cruz Biotechnology, Inc. Santa Cruz CA, USA) and anti-phospho-ERK (1∶1000, sc-7383, Santa Cruz Biotechnology, Inc. Santa Cruz CA,USA). Membranes were incubated with corresponding horseradish peroxidase-conjugated secondary antibody (1∶10,000) and were developed using a chemiluminescent reagent (ECL detection reagent Amersham Biosciences). Developed signals were recorded on X-ray film (Fujifilm) for densitometric analysis (Scion Image Frederick, Maryland USA).

### Quantification of renal mTOR, Akt and TGFß1 by quantitative Real-Time PCR (qRT-PCR)

Total RNA was obtained following the Trizol manufacturer's instructions (Invitrogen, Carlsbad). The RNA used for the study had a 28 s/18 s ratio between 1.8 and 2.0. Total RNA was reverse-transcribed as follows: 2 µg of RNA were incubated with 1 µl of 50 µM random hexamers followed by RNA denaturalization. Then, 5× reaction buffer, 0.4 µl of 100 mM dNTP mix, and MMLV retrotranscriptase 200 u/µl (Ecogen, Langhorne, PA, USA) were added in a final volume to 20 µl and the reaction was incubated during 5 min at 25°C, followed by 30 min at 42°C and 5 min at 85°C to stop the reaction. qRT-PCR was performed using 5 µl of cDNA, primers and the TaqMan probe for Akt (Rn-00583646_m1), mTOR (Rn-00571541) and TGFβ_1_ (Rn-00572010_m1). It is directed to locus gene NM 021578 that codifies for the translated protein NP_067589, precursor of total TGFβ_1_ (A pplied Biosystems, CA, USA), and the ABI Prism 7700 Sequence Detector. The level of target gene expression was determined using the ΔCt method as described [50] normalized to the Actin control (Applied Biosystems, Madrid, Spain). Results were expressed as ‘many fold of the unknown sample’ relative to the reference value (sham group). Triplicates were done in each experiment. Results are expressed as mean ± SEM.

### Statistical analysis

All data were expressed as Mean ± SD. One way analysis of variance and post hoc with Student-Newman–Keuls test was used to determine the statistical significance. Statistical significance was defined as P value less than 0.05.
